# Changes in protein structure at the interface accompanying complex formation

**DOI:** 10.1107/S2052252515015250

**Published:** 2015-10-16

**Authors:** Devlina Chakravarty, Joël Janin, Charles H. Robert, Pinak Chakrabarti

**Affiliations:** a Department of Biochemistry, Bose Institute, P-1/12 CIT Scheme VIIM, Kolkata 700 054, India; b IBBMC, CNRS UMR 8619, Universite Paris-Sud 11, Orsay, France; c CNRS Laboratoire de Biochimie Theorique, Institut de Biologie Physico-Chimique (IBPC), Universite Paris Diderot, Sorbonne Paris Cité, 13 Rue Pierre et Marie Curie, 75005 Paris, France

**Keywords:** protein–protein interactions, protein flexibility, disorder–order transition, bound and unbound protein forms, interface area, crystallographic temperature factor, secondary structure, bioinformatics, molecular recognition

## Abstract

Protein interactions are essential in all biological processes. The changes brought about in the structure when a free component forms a complex with another molecule need to be characterized for a proper understanding of molecular recognition as well as for the successful implementation of docking algorithms. Here, unbound (U) and bound (B) forms of protein structures from the Protein–Protein Interaction Affinity Database are compared in order to enumerate the changes that occur at the interface atoms/residues in terms of the solvent-accessible surface area (ASA), secondary structure, temperature factors (*B* factors) and disorder-to-order transitions. It is found that the interface atoms optimize contacts with the atoms in the partner protein, which leads to an increase in their ASA in the bound interface in the majority (69%) of the proteins when compared with the unbound interface, and this is independent of the root-mean-square deviation between the U and B forms. Changes in secondary structure during the transition indicate a likely extension of helices and strands at the expense of turns and coils. A reduction in flexibility during complex formation is reflected in the decrease in *B* factors of the interface residues on going from the U form to the B form. There is, however, no distinction in flexibility between the interface and the surface in the monomeric structure, thereby highlighting the potential problem of using *B* factors for the prediction of binding sites in the unbound form for docking another protein. 16% of the proteins have missing (disordered) residues in the U form which are observed (ordered) in the B form, mostly with an irregular conformation; the data set also shows differences in the composition of interface and non-interface residues in the disordered polypeptide segments as well as differences in their surface burial.

## Abstract 2

To understand molecular recognition, one needs to characterize the conformational changes that take place when a free (unbound) molecule forms a complex with another protein. An analysis of 281 protein components is presented, delineating such changes in terms of accessible surface area, secondary structure, crystallographic *B* factors and disorder-to-order transitions.

## 1. Introduction   

Protein–protein recognition plays a crucial role in many biological processes, including DNA replication, protein degradation, signal transduction and metabolic processes (Stites, 1997 ▸). Interaction with small molecules, nucleic acids and other proteins takes place through binding at specific sites. Protein–protein interactions have distinct characteristics, such as direct physical contact, surface complementarity and a specific, well defined interface. Protein structure, dynamics and function are interdependent. Relating structure to dynamics and function is essential in understanding molecular-recognition processes (Boehr *et al.*, 2009 ▸; Mittag *et al.*, 2010 ▸; Tompa & Fuxreiter, 2008 ▸). Factors such as hydrophobicity and the specific location of residues capable of forming hydrogen bonds and electrostatic interactions help to distinguish the interface from the rest of the surface (Janin *et al.*, 2008 ▸). Jones and Thornton, amongst others, have discussed the features of interface patches, for instance residue propensity, planarity, surface accessibility and protrusion, that make them different from the rest of the protein surface (Jones & Thornton, 1995 ▸, 1997 ▸). Conservation of interface residues and their clustering can also be used as a discriminating factor (Guharoy & Chakrabarti, 2010 ▸). Interface residues undergo more significant conformational changes than other surface residues and have evolved to retain the specificity of their interactions (Rajamani *et al.*, 2004 ▸). Although many proteins interact as quasi-rigid bodies, undergoing very little conformational change as they form complexes, in many cases the conformational change is significant and may or may not be restricted to the interface region (Swapna *et al.*, 2012 ▸). Proper integration of these changes is important for estimating the binding affinity between two proteins from structural data (Janin, 2014 ▸), yet incorporating them is the main bottleneck in the development of robust protein–protein docking algorithms (Aloy *et al.*, 2005 ▸; Bonvin, 2006 ▸). There have already been attempts to decipher the changes associated with the transition of the free (or unbound) form of a molecule to the bound form in the complex, for example involving side-chain conformations (Guharoy *et al.*, 2010 ▸; Ruvinsky *et al.*, 2011 ▸).

Analyses of protein–protein interfaces are usually performed on complexes that are available as crystal structures. However, proteins are dynamic and exist in ensembles of interchanging structures. Binding to small molecules, nucleic acids or other proteins leads to shifts in the populations of these conformers; the term conformational change is a shorthand for such functional shifts. The affinity in particular depends on both the unbound-state and bound-state ensembles. To better understand the changes brought about by association, we have used the protein–protein binding-affinity benchmark, which is a nonredundant set of 144 complexes for which high-resolution structures are available for both the complexes and their unbound components and for which dissociation constants have been measured using biophysical techniques (Kastritis *et al.*, 2011 ▸). We have recently looked at the changes in accessible surface area of interface atoms in pairs of unbound (U) and bound (B) forms of proteins (Chakravarty *et al.*, 2013 ▸); in this work, we extend these analyses and also study the changes in secondary structures, temperature factors (*B* factors) and disorder-to-order transitions.

## 2. Materials and methods   

We used the Protein–Protein Interaction Affinity Database (Kastritis *et al.*, 2011 ▸) containing 144 complexes along with the corresponding unbound structures, except for seven antibody–antigen complexes for which the unbound structure is not known. We thus considered 281 bound–unbound structure pairs. The unbound structure is designated U and the bound structure (isolated from its partner component) is designated B. The bound form of the component in the presence of its partner (*i.e.* in complex) is designated C. *EMBOSS* (Rice *et al.*, 2000 ▸) was used to perform the local alignment (using the Smith–Waterman algorithm) of the polypeptide chains constituting U/B pairs; 249 had a sequence identity of ≥96%, with the rest having values in the range 90–95%. Based on the sequence alignment, the interface residues as seen in the complex were mapped onto those in the unbound state using *ProFit* (McLachlan, 1982 ▸) and *Biopython* (Cock *et al.*, 2009 ▸). Amino acids differing between the bound and unbound protein sequences (Supplementary Table S1*a*), and positions at which data are missing owing to order–disorder transitions in the two PDB files under consideration (Supplementary Table S1*c*), were excluded from structural analysis. There are 27 structures with different residue names in U and B affecting 40 residues. Any modified residues (Supplementary Table S1*b*) were manually edited to match the natural amino acid. Interface atoms were identified as those losing more than 0.1 Å^2^ of surface area upon complex formation (B to C; Chakrabarti & Janin, 2002 ▸). *NACCESS* (Hubbard, 1992 ▸) was used for the calculation of solvent-accessible surface area (ASA). As discussed in Chakravarty *et al.* (2013 ▸), one has to consider the ambiguity in atom labels (especially of aromatic residues) while calculating the surface area buried in going from U to B. Hydrogen bonds were assigned using *HBPLUS* (McDonald & Thornton, 1994 ▸) with default geometrical parameters. Secondary structures were determined using *DSSP* (Kabsch & Sander, 1983 ▸).

In the affinity data set, 17 unbound structures were determined by NMR, of which 13 have multiple models or conformations. For each of them the first model was used. However, in a control calculation, the surface parameters calculated using all of the models were found to be essentially the same as those employed in the reported calculations.

### 2.1. Terms and equations used to describe the changes in the interface  

(i) ΔASA = [ASA(B) – ASA(U)], where ASA(B) is the solvent-accessible surface area of the mapped interface atoms in the bound state and ASA(U) is the value in the unbound state. In this calculation only the interface atoms present in both states were used. This is distinct from the buried surface area, BSA = [ASA(B) – ASA(C)], calculated in the standard way using all of the interface atoms.
(ii) δA = ΔASA/ASA(B) is the difference in ASA relative to the total value in the bound state.
(iii) bf_*r*_′ = [bf_*r*_ − μ(bf)]/σ(bf), where bf_*r*_ is the average *B* factor of the C, C^α^, O, N and C^β^ atoms of residue *r* (C, C^α^, O and N for Gly) and μ(bf) and σ(bf) are the mean and standard deviation of the *B* factors for that chain, respectively. The normalized bf_*r*_′ values were used to derive the averages over the interface, surface and core and rim regions of the interface (Chakrabarti & Janin, 2002 ▸).
(iv) *D* = 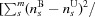 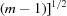; the Euclidean distance *D* was used to quantify the change in *n_s_*, the percentage composition of the secondary-structure type *s* of interface residues, between the bound (*n* ^B^_*s*_) and the unbound (*n* ^U^_*s*_) forms; the *m* = 4 secondary-structure types are defined as helix, strand, turn and coil.
(v) The Euclidean metrics, Δ*b*, for the *B* factors of residues in different states/structural regions were calculated in a similar way, Δ*b* = 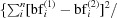 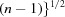, where *n* represents the number of amino-acid types and bf_*i*_^(1)^ and bf_*i*_^(2)^ are the scaled *B* factors of residue type *i* in states 1 and 2, respectively. The states that were compared were interface, non-interface, bound and unbound.

## 3. Results   

### 3.1. Change in the ASA of interface atoms on going from the U state to the B state   

**Figure 1:**
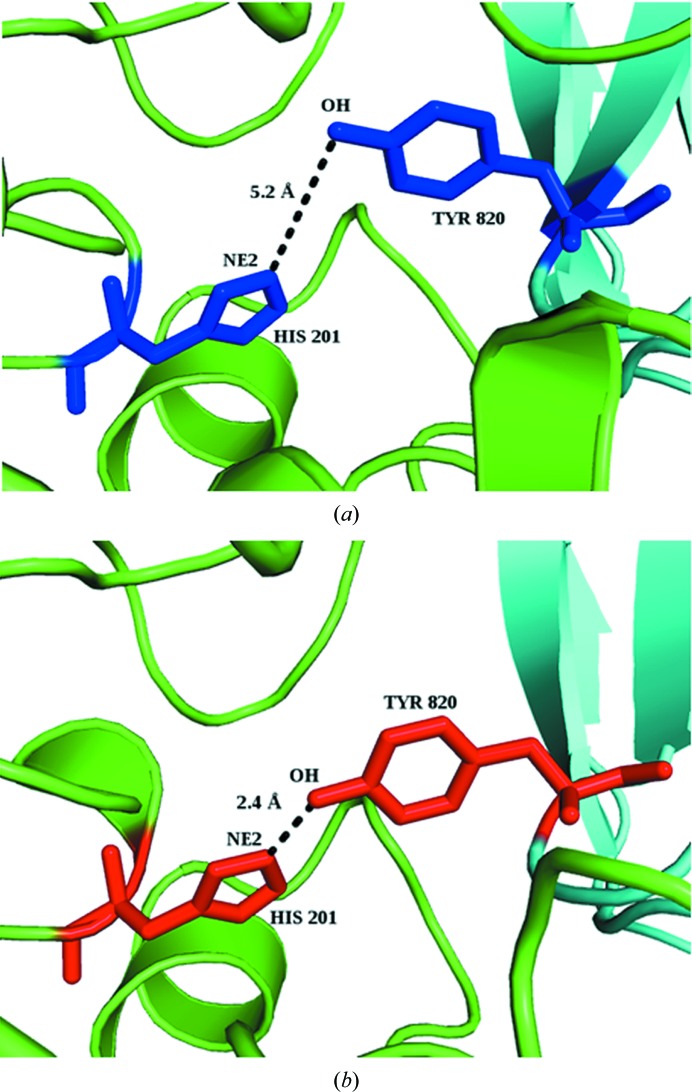
Hydrogen-bond geometries (distances shown) in α-amylase (green) and tendamistat (cyan) between His201 NE2 and Tyr820 OH for (*a*) the pseudo-complex and (*b*) the experimental complex [PDB entry 1bvn (Wiegand *et al.*, 1995 ▸); PDB entries 1pig (Machius *et al.*, 1996 ▸) and 1hoe (Pflugrath *et al.*, 1986 ▸) are the U forms]. ΔASA for the participating atom and all of the interface atoms of the residues are −0.6 and −3.2 Å^2^, respectively, for His, and 4.2 and 15.5 Å^2^, respectively, for Tyr.

Previously, we had shown that on going from the U form to the B form the interface atoms undergo an increase in accessible surface area (ASA), leading to a positive δA value (Supplementary Fig. S1; mean = 3.3 ± 9.2%), which is the result of conformational changes taking place at the interface (Chakravarty *et al.*, 2013 ▸). (As a control, we checked the variation of the ASA of free surface residues, which show only an insignificant increase, with a mean value of 0.90 ± 6.06%.) Considering the whole residue, which includes non-interface atoms, the increase can still be seen (1.3 ± 8.03%) but is smaller than that exhibited by the interface atoms alone. The ASA increase reflects what might be called a ‘partner attraction effect’: interface atoms are extended in the bound state to optimize contact with the binding partner. In addition to maximizing van der Waals interactions, the increase in the ASA of interface atoms could also be the result of optimizing interchain hydrogen-bond geometry. As a simple quantification of this, we used structures for which the combined r.m.s.d. for the U-to-B change for the two components (I_r.m.s.d. according to Kastritis *et al.*, 2011 ▸) is <1 Å. For these 59 cases we generated the pseudo-complex by superimposing the two U forms onto the corresponding B structures. The average number of hydrogen bonds in the pseudo-complex is 3.7 ± 2.5, whereas in the real complex it is 8.0 ± 3.7, a 45% increase. An example of the local adjustment of the two U structures leading to the formation of a hydrogen bond in the complex is shown in Fig. 1 ▸: the structural rearrangement pulls out the Tyr residue such that there is a net gain in ΔASA.

**Figure 2:**
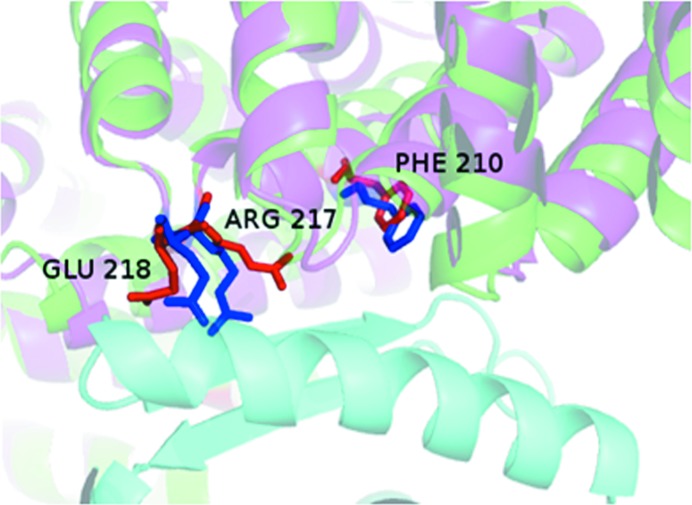
The complex between the core domain of HspBP1 and the Hsp70 ATPase domain, an example of the change in the position of interface residues (stick representation; red in the B form and blue in the U form). Protein chains are shown in cartoon representation in green for the B form (PDB entry 1xqs) and in pink for the U form (PDB entry 1xqr) of the core domain of HspBP1 (Shomura *et al.*, 2005 ▸) containing the labelled interface residues; the other component (the Hsp70 ATPase domain) in the B form is shown in cyan. ΔASA = −175 Å^2^ and δA = −10%. The ΔASA values for the interface atoms of the residues shown are −43 Å^2^ for Arg217, −20 Å^2^ for Glu218 and −16 Å^2^ for Phe210.

**Figure 3:**
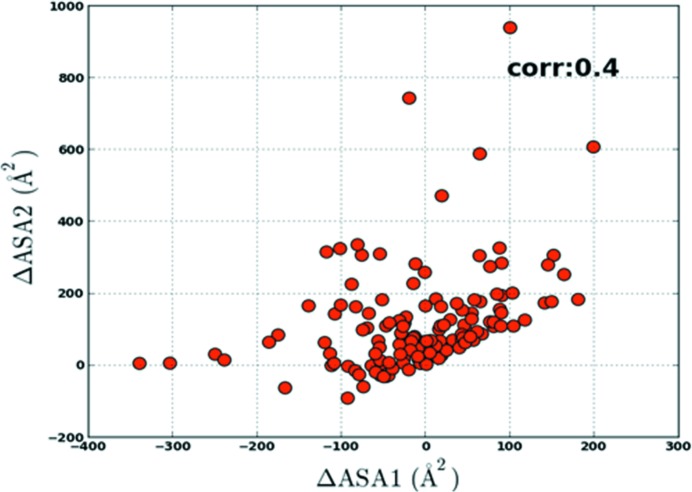
Plot of ΔASA of the interface atoms separated into the two components for each complex. The greater of the two values is labelled ΔASA2 and the lesser ΔASA1.

While the majority of complexes show an increase in ASA, 31% (88 of the 281 components) have a negative δA value, indicating that the interface atoms are pulled back into the structure to facilitate the interaction with the incoming partner molecule: a ‘partner accommodation’ effect. Fig. 2 ▸ shows such an example with a δA value (−10%) from the opposite side of the distribution. It is seen that for the core domain of the HspBP1 protein the effect of binding has been to pull the interface atoms, which were extended into the solvent in the U form, towards itself to allow a closer approach by the partner molecule (the Hsp70 ATPase domain). While the two component contributions in the complex are weakly correlated (Fig. 3 ▸), we note that the proportions of complexes in which the ΔASA contributions of the two partner proteins are both positive (65 complexes; 47%), both negative (15 cases; 11%) and mixed positive and negative (57 complexes; 42%) are consistent with a simple statistical model of independent component contributions (*p* ^2^_+_ = 47%, *p* ^2^_−_ = 10% and 2*p*_+_ *p*_−_ = 43% for *p*_+_ = 0.69 and *p*_−_ = 0.31). Thus, the ‘partner accommodation effect’ does not usually operate simultaneously on both components, and complex formation is usually accompanied by the ‘partner attraction effect’. ΔASA has a poor correlation with interface r.m.s.d. and BSA (Supplementary Fig. S2), indicating it to be essentially independent of the size of the interface or the root-mean-square deviation of the interface atoms.

### 3.2. Changes in secondary structure   

**Figure 4:**
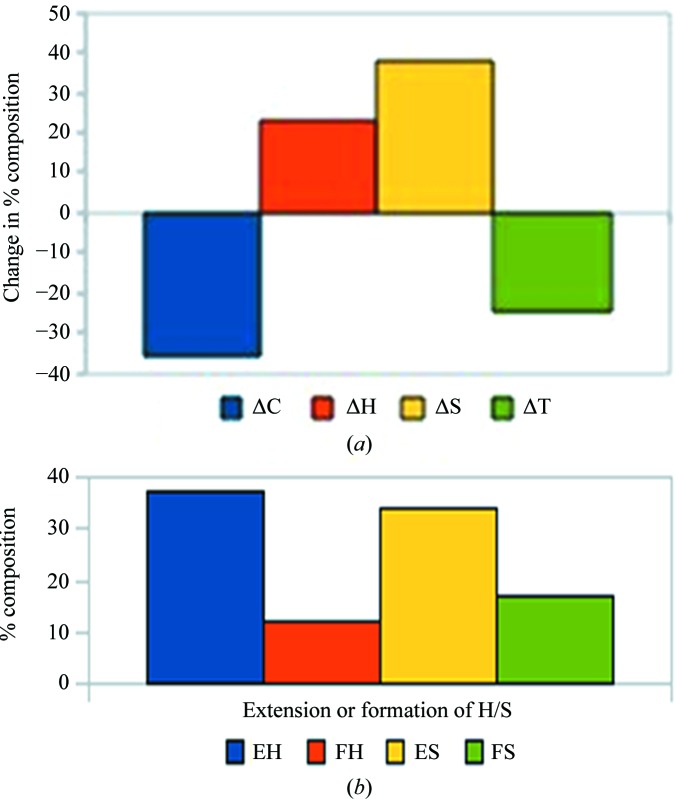
Secondary-structural changes during the U-to-B transition. (*a*) The change in percentage composition between the two states (B – U) for the secondary-structural elements (helix, H; strand, S; turn, T; irregular, C) for the cases with Euclidean distances between the two sets of compositions of >5. (*b*) Percentage composition of 224 residues showing the C/T to H/S transition, categorized into the extension of an already existing helix/strand (EH and ES) or the formation of a new helix/strand (FH and FS).

**Figure 5:**
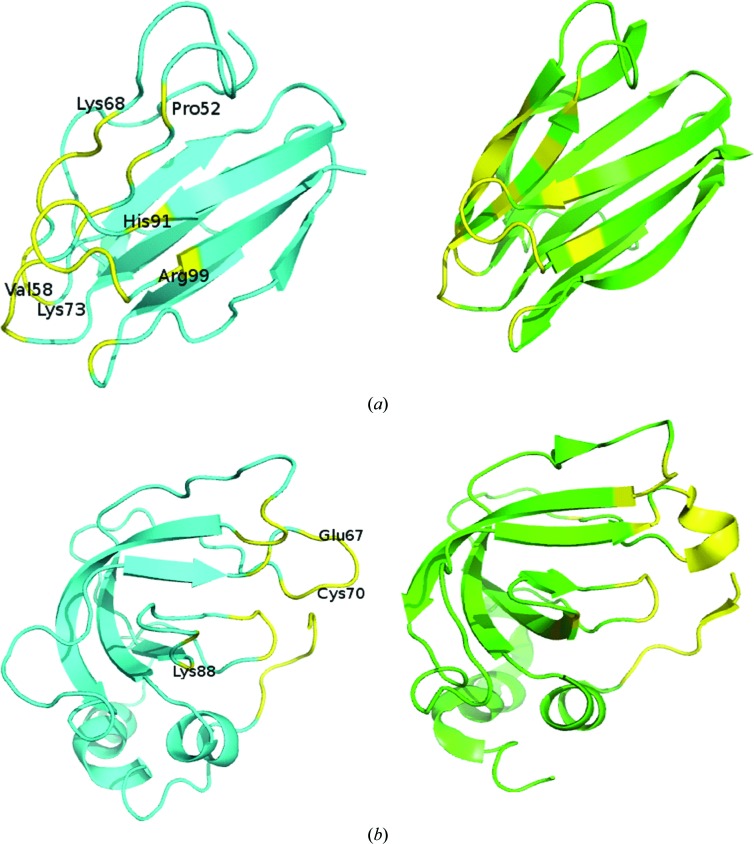
Examples showing changes in secondary-structural elements (left panel, U; right panel, B). Stretches in the interface are in yellow. (*a*) Amicyanin (PDB entry 2rac; Zhu *et al.*, 1998 ▸) in complex (PDB entry 2mta; Chen *et al.*, 1994 ▸) with methylamine dehydrogenase exhibits the formation of two antiparallel β-strands (Pro52, Asn54, His56 and Val58 in one, and Lys68, Gly69, Pro70, Met71 and Lys73 in the other) and N-terminal (Arg99) and C-terminal (His91) extension of two other strands. (*b*) Metalloproteinase inhibitor 1 (PDB entry 1d2b; Wu *et al.*, 2000 ▸) in complex (PDB entry 2j0t; Iyer *et al.*, 2006 ▸) with MMP1 intersitial collagenase displays the formation of a small helix (Glu67, Ser68, Val69 and Cys70) and C-terminal (Lys88) extension of a strand.

The change in the percentage composition of secondary-structural elements for the U to B transition was calculated, and 76% cases (213 of 281) showed some change. To restrict the analysis to meaningful changes, we computed the Euclidean distance (*D*) between the compositions of the four structural elements. The average value of *D* is 5.6 (±5.4), and we used structural pairs with *D* > 5 (134 cases) to understand the structural changes accompanying complex formation (Fig. 4 ▸ *a*) [the histograms for *D* > 10 and *D* > 15 (Supplementary Figs. S3*a* and S3*b*) look very similar]. It can be seen that complex formation leads to an increase in helical and strand content (especially the former) at the expense of irregular (and to some extent turn) regions in the structure. 91 structural pairs show an irregular/turn (C/T) to helix/strand (H/S) transition, affecting 75 helices and 81 strands, corresponding to 34% of helices and 38% of strands, respectively, of these structural elements in the B form of the proteins. These cases have an average *D* value of 7.8 ± 4.9, with 224 residues changing conformation. The majority of these (161 cases) are involved in the extension of an already existing helix or strand (Fig. 4 ▸ *b*). Cases of extension seem to marginally favour the C-terminal end of helices and the N-terminal end of strands (Supplementary Fig. S3*c*). The residues located in the interface core (108 of 224; 48%) and rim (52%) are affected equally, among which Arg, Glu, Ser and Tyr are those more frequently involved in the transition from C/T to H/S. Two representative examples showing secondary-structural changes are presented in Fig. 5 ▸.

### 3.3. Analysis of missing residues in the unbound form   

95 proteins of the 281 have one or more interface atoms missing in the crystal structure of the U state that are present in the B state. 46 proteins (16%) have interface residues missing in the unbound form (on average four missing residues per component). We will refer to these atoms and residues as ‘missing’ even though they are clearly present in the bound form. Missing atoms constituted ∼4% of the total interface atoms in the data set and 12.5% for the 95 structures, with the most extreme being MAPKAP kinase 2 (PDB entry 3fyk; Anderson *et al.*, 2009 ▸), in which 52% of the interface atoms (39 of 80 interface residues) are missing. Usually the interface and non-interface residues are interspersed in a given stretch of missing residues, and there are 34 such cases (12%) with two or more missing residues, the sequences of which can be seen in Supplementary Table S2. Such regions undergo a disorder-to-order transition upon binding to their interacting partner, and have special importance in elucidation of the structure–function relationships of proteins (van der Lee *et al.*, 2014 ▸). Interestingly, in more than half of the cases the missing segment is at the polypeptide chain termini.

The statistics for the missing residues in the U form are provided in Table 1 ▸. For all of the missing stretches of amino acids given in Supplementary Table S2, two values are reported: one concerning only the interface residues and the other only the non-interface residues. The composition of missing residues is similar to that of the interface as a whole, although aromatic residues (Phe, Tyr and Trp) seem to have a lower tendency to be disordered. Relative to the total number of missing interface residues, among the charged residues the longer ones are found in greater numbers (Glu > Asp, Arg > Lys or His). Interestingly, the composition of nonpolar interface residues (Ala, Leu and Ile) is also on the higher side. Thus, the hydrophobic effect would appear to have a role in determining which residues in the disordered regions contribute to binding. It may be noted that intrinsically disordered proteins are known to expose their few hydrophobic residues for interaction with the partner (Mészáros *et al.*, 2007 ▸).

We also analyzed the secondary structures adopted in the bound form by these missing residues. In general, >50% of the missing interface residues adopt an irregular conformation in the bound state; next in level of occurrence are helices and turns, with strands seeming to be the least favoured. Considering interface residues located in the terminal peptide segments only, one observes a slightly higher tendency to adopt an irregular conformation (65%, as opposed to 20% in T and 15% in H). Met and Leu assume a helical conformation in greater percentages, as can be expected from the generally higher preference of these residues for this type of secondary structure.

Of the missing residues, 49% become core interface residues (those which have atoms fully buried in the interface); however, these contribute 70% of the BSA; the BSA values of core residues missing in U exceed those of the rim with a *P* value of 0.01. These observations are in conformity with a previous report (Chakrabarti & Janin, 2002 ▸). In the 46 proteins missing one or more interface residues, the latter contribute 17% of the BSA in the bound state (190 ± 278 Å^2^ of 1017 ± 568 Å^2^); however, the distribution is rather large, ranging from ∼57% in the MAPK-activated protein kinase 2 (MK2) part of the assembly formed with p38 (PDB entries 3fyk and 2oza; Anderson *et al.*, 2009 ▸; White *et al.*, 2007 ▸) to 0.1% in the complex formed by Ran-specific GTPase-activating protein with GTP-binding nuclear protein RAN (PDB entries 1yrg and 1k5d; Hillig *et al.*, 1999 ▸; Seewald *et al.*, 2002 ▸), with 39 and one residues, respectively, missing in the U state.

In the 95 structures with missing atoms, each missing atom contributes 11.5 ± 6.8 Å^2^ to the BSA in the bound state, while the remaining (‘non-missing’) atoms each contribute 9.4 ± 1.5 Å^2^ (*P* = 3 × 10^−21^ for the two populations with 977 and 6945 atoms, respectively). Thus, at the local level the interface elements that undergo a disorder-to-order transition upon forming the complex bury somewhat more surface than other atoms in the interface. However, at the database level the effect is more marked; structures for which no interface residues are missing in the U form bury on average 786 ± 336 Å^2^ in the complex, while structures with one or more interface residues missing bury more: 1017 Å^2^, as given above (*P* = 0.005). Indeed, when calculated for the structures missing five residues or more (13 structures in all), surface burial is larger still: 1507 ± 795 Å^2^ (*P* = 0.003). The presence of missing residues is thus seen to be associated with a larger degree of surface burial upon forming the complex, similar to systems undergoing a conformational change upon association (Kastritis *et al.*, 2011 ▸); both effects presumably indicate compensation of the corresponding free-energy penalty.

**Figure 6:**
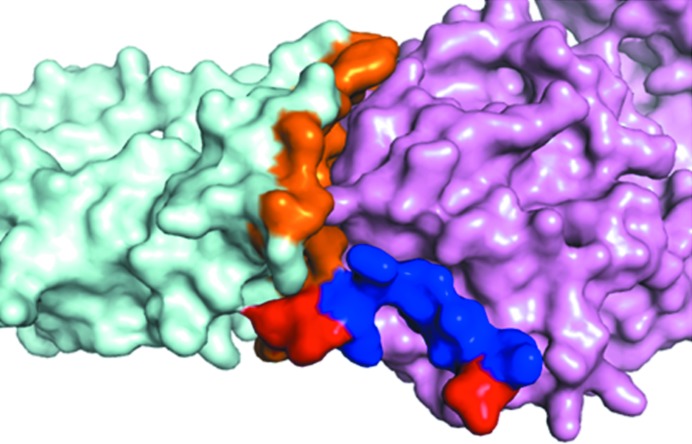
The structure of the interface formed in human tissue inhibitor of metalloproteinases 2 when it forms a complex with type IV collagenase (PDB entry 1gxd; Morgunova *et al.*, 2002 ▸); the inhibitor is denoted in cyan and the enzyme in violet. Surface representations of the proteins are displayed. The U state (PDB entry 1br9; Tuuttila *et al.*, 1998 ▸) is not shown here. The interface residues are split into two categories: the residues missing in the unbound structure are in blue and those seen in both the U and B forms are in orange. The missing segment (183–192) is composed of both non-interface residues (shown in red) and interface residues (blue). The missing residues contribute 504 Å^2^ to the BSA of 1268 Å^2^ of the inhibitor.

The total interface in a complex is made up of contributions from both components, the BSA values of which are generally similar but not equal. It is of interest to study the contribution of a specific residue not only to the BSA of its own component (‘parent’), but also to that of the partner component owing to their interaction. Overall, the missing residues in a given component contribute 155 ± 270 Å^2^ to the BSA of the parent and nearly the same (156 ± 246 Å^2^) to that of the partner. However, in those cases for which missing residues contributed more than 200 Å^2^ to the parent (13 structures, missing nine residues per structure on average), the contribution to the partner was smaller on average by 34 ± 50 Å^2^. It may be mentioned in connection that the BSA values of the interacting proteins are normally nearly identical; in the case of protease–inhibitor complexes, however, the convex nature of the inhibitor surface fitting into the concave active site results in its BSA exceeding that of the enzyme in the ratio 54:46 (Lo Conte *et al.*, 1999 ▸). Figs. 6 ▸ and 7 ▸ provide two illustrations of a missing segment and the structure (mostly of irregular conformation) adopted in the B form. The example in Fig. 6 ▸ is a case in which the gain in BSA from missing residues in the parent molecule exceeds that in the partner by 139 Å^2^. The asymmetric nature of the BSA values for the two sides is owing to the better fitting of the disordered residues into the grooves and crevices of the more ordered interacting partner. Favourable interactions arising from the burial of these residues should also help to compensate for the entropic loss of ordering them.

The residue composition does not change much if we consider the non-interface residues in the missing stretches of amino acids (Table 1 ▸); along with the charged residues (Glu, Asp, Arg, Lys), Ser, Thr and Gly are seen to occur in large numbers as well. This is in accordance with what is observed in intrinsically disordered proteins, which are enriched in charged and polar amino acids and depleted in bulky hydrophobic groups (van der Lee *et al.*, 2014 ▸). The missing stretches exhibit similar features as the interface residues within them, usually taking up turn or irregular conformations in the bound structure.

### 3.4. Comparison of *B* factors   

The crystallographic *B* factor (temperature factor or atomic displace­ment parameter) is a measure of the oscillation of an atom around its mean position owing to thermal motion and positional disorder. Normalized *B* factors have been used to compare structures (Parthasarathy & Murthy, 2000 ▸). It has been recognized that residues in the interface have lower *B* factors than those in the protein exterior (Jones & Thornton, 1995 ▸), suggesting that residues participating in protein–protein interactions are less flexible than those on the free surface. This inference was based on an analysis of complex structures only. However, a subsequent comparison of 57 monomeric structures with their bound homologues (>70% sequence identity) indicated that even in the unbound state the distribution of *B* factors is somewhat lower for the interface than for the rest of the surface (Neuvirth *et al.*, 2004 ▸). Along these lines, we have compared the interface and the surface residues for the bound as well as the unbound structures.

**Figure 8:**
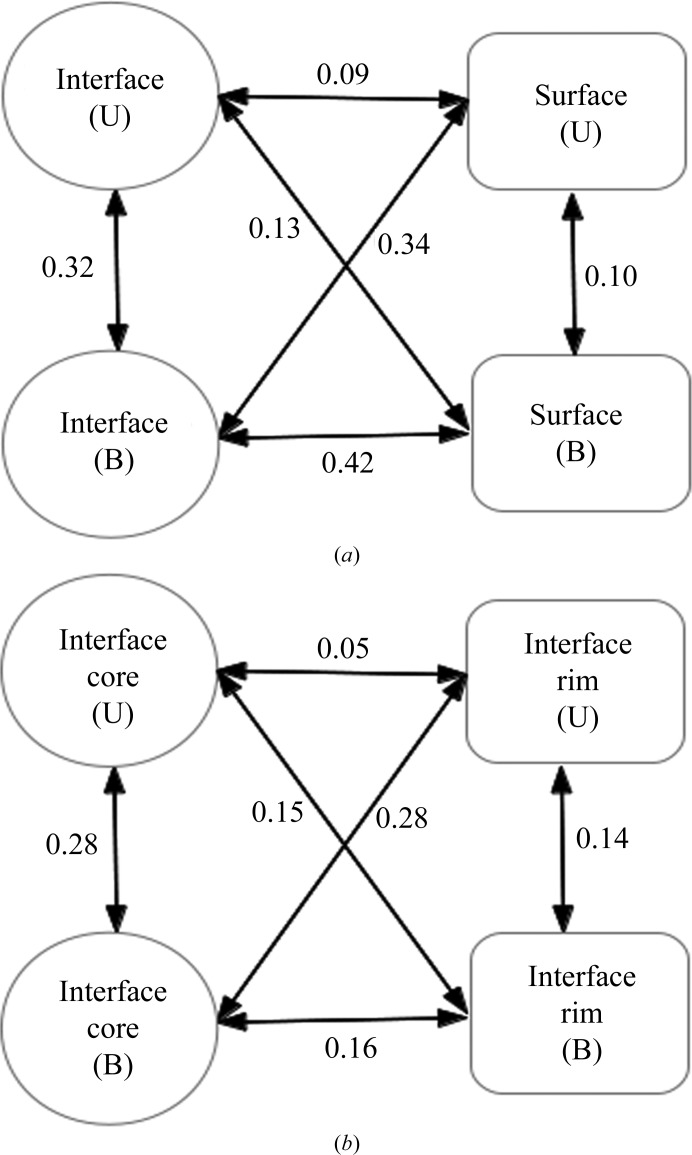
Euclidean distances involving *B* factors (*a*) between interface and surface regions (enumerated in Supplementary Table S3) and (*b*) between interface rim and core regions (Supplementary Table S4) in the U and B states.

The scaled mean *B* factor of the backbone atoms C, C^α^, O and N (along with C^β^ for non-Gly residues) were calculated along with the average values for each residue type in the interface and the surface regions for both the U and B states. As expected, the average *B* factor was observed to be greater for the surface compared with the interface in the B structures (*P* value < 2 × 10^−16^; Supplementary Table S3, Fig. 8 ▸ *a*). Indeed, the normalized *B* factors for all of the residues in the interface are negative (below the average value for all of the residues in the structure). In contrast, in the unbound structures the interface residues mostly have positive values and, as expected, the values are higher than those observed in the bound interface. Thus, on going from the the U state to the B state the interface residues exhibit a drastic reduction in *B* factor. Although the changes are not as strong, overall the opposite trend was observed for the surface residues (*P* value = 0.04). Again applying a Euclidean metric, here defined using the average *B* factors of amino-acid residues in the two regions of the protein structures and in the two states (Supplementary Table S3), we find that the maximum changes occur in the interface region as the complex is formed and between the interface and the surface regions in the complex. Overall, the *B* factors in U are quite similar between the interface and the surface. Interestingly, however, hydrophobic residues (notably the aromatic residues) tend to be more flexible at the interface compared with the surface in the U state, while the opposite seems to be the case for polar residues. Grouping Ile, Leu, Met, Phe, Trp and Tyr as nonpolar and Arg, Asn, Cys, Gln, Glu, Gly, His, Lys, Ser and Thr as polar, the difference in *B* factors is significant (the *P* values are 0.05 and 0.048, respectively). It has been noted that the δA values are higher (>4%) for all of the nonpolar residue types (Chakravarty *et al.*, 2013 ▸). The higher flexibility in the U state of the nonpolar residues in the region that would constitute the interface (in B) may thus predispose them to conformational changes accompanying complex formation.

The interface residues were further divided into core and rim regions (Chakrabarti & Janin, 2002 ▸), and *B* factors were also compared between these two regions in the U and B states (Supplementary Table S4). The reduction in *B* factors is more pronounced in the core region between the two states, which can also be seen from the Euclidean distance between them (Fig. 8 ▸ *b*); the rim residues show a smaller difference between the two forms. This is also reflected in the *P* values (2 × 10^−16^ for the core and 7.602 × 10^−9^ for the rim). An illustration of these results for a representative protein is shown in Supplementary Fig. S4. Comparing the core–rim demarcation in Supplementary Fig. S4(*e*) with the distribution of *B* factors in the B form (Supplementary Fig. S4*b*), one can see considerable matching for the core (dark blue). There is very little resemblance to the *B* factors observed for the U form (Supplementary Fig. S4*d*). Indeed, there is no significant difference overall between the core and rim residue *B* factors in the U form (*P* value = 0.97). This is in contrast to the results from molecular-dynamics simulations, which had indicated a lesser fluctuation of the core residues even when the binding partner is absent (Smith *et al.*, 2005 ▸).

## 4. Discussion   

Computation of the accessible surface area (ASA) has been very useful in the identification of interface residues (Janin *et al.*, 2008 ▸) and in segregating the interface into core, rim and support regions (Chakrabarti & Janin, 2002 ▸; Levy, 2010 ▸). It has been used to predict the magnitude of binding-induced conformational changes from the structures of either monomeric proteins or bound subunits (Marsh & Teichmann, 2011 ▸). Here, ASA has been used to compare the interface atoms in the unbound and bound states of a protein. Conformational changes brought about by protein–protein interaction are often discussed in the context of ‘induced fit’, which however fails to capture the sense of the change observed in the ASA calculations. Two terms have thus been coined here to distinguish between the increase in the ASA of interface atoms upon complex formation and their decrease: ‘partner attraction’ and ‘partner accommodation’ effects, respectively. The former is observed to dominate, although clear examples of the latter are also observed; both are examples of induced fit in the broad sense. In a complex, the interface atoms tend to make fewer contacts within their component as they interact with the other component; it is as if the atoms are pulled out of the parent molecule for optimum binding to the partner molecule (Chakravarty *et al.*, 2013 ▸). It has been suggested that the change in side-chain conformation of interface residues may lead to an increase in the relative solvent-accessible surface area on complexation (Ruvinsky *et al.*, 2011 ▸). However, rather than at the level of the residue as a whole, we find that the increase in ASA is more at the level of interface atoms. ΔASA seems to be independent of the overall size of the interface or of whether the molecule binds as a rigid body or exhibits conformational changes.

Unlike intrinsically disordered proteins (IDPs) made of entirely disordered sequences that do not adopt any tertiary structure in the uncomplexed state, 16% of the proteins considered here contain both structured and disordered regions, which are seen to contribute to protein–protein interaction and thereby possibly facilitate the regulation of cellular processes (van der Lee *et al.*, 2014 ▸; Dyson & Wright, 2002 ▸; Dunker *et al.*, 2001 ▸; Fong *et al.*, 2009 ▸; Ruvinsky *et al.*, 2011 ▸). Features of disordered regions revealed in this work, such as amino-acid preferences, the amount of ASA buried in complexation *etc.*, may be general characteristics of IDPs (and intrinsically disordered regions; IDRs), especially in proteins which function as effectors (van der Lee *et al.*, 2014 ▸; Guharoy *et al.*, 2015 ▸).

Besides the ASA, parameters such as hydrogen-bonding patterns, secondary-structure changes and *B* factors can also be very useful in discerning the interface from the surface. We have seen that in proteins that undergo secondary-structure changes upon the U-to-B transition the most common are extension of the existing helices and strands at the expense of turns and irregular regions (Fig. 4 ▸). By defining disordered residues as those with missing coordinates in the crystal structure, one discerns features of disorder-to-order transitions of IDPs or IDRs that play a role in binding. Overall, in going from the U state to the B state proteins adopt a more ordered and regular structure: however, regions showing a disorder-to-order transition tend to assume more irregular secondary structures in the B state, while parts which were already ordered in the U state tend to shift towards more regular secondary structures, if they change at all. The ordering of missing residues upon complex formation adds an entropic penalty to the association reaction, which appears to be compensated in part by a greater degree of surface burial in complexes with missing residues, similar to what has been observed for conformational changes (Kastritis *et al.*, 2011 ▸).

Previous studies have demonstrated that in protein–protein complexes (B form) the *B* factors of the interface residues are lower than those of the surface residues (Jones & Thornton, 1995 ▸; Liu *et al.*, 2010 ▸). In the bound interfaces, we also see that the interface core is significantly less mobile than the rim. In the unbound interfaces, the nonpolar residue flexibility appears to actually be somewhat higher than in the non-interface surface, which may be related to these residue types contributing somewhat more to the increase in ASA upon complex formation. Overall, however, the marked *B*-factor differences seen in the B form are of course not present in the U form, in which the interface region is solvent-exposed and mobile. *B* factors have been used in constructing support vector machines (SVMs) and other popular classifiers to identify protein–protein interaction sites and to distinguish biological interfaces from crystal contacts (Liu *et al.*, 2010 ▸, 2014 ▸; Neuvirth *et al.*, 2004 ▸). Our results point to limitations in using *B* factors for the identification of the binding site, especially if one is focusing on the U structure, which is more relevant than the B form in the context of protein–protein complex prediction. Likewise, along with other interactions, hydrogen bonding has been used as a feature for predicting protein–protein interaction sites among targets of homologous proteins (Maheshwari & Brylinski, 2015 ▸); however, the latter is necessarily based on complex structures, which as Fig. 1 ▸ shows cannot be properly reproduced by the unbound form even for proteins that are considered to behave as quasi-rigid bodies when forming the complex.

## 5. Conclusions   

Nearly 90% (122 of 137) of the protein–protein complexes considered in this study show an increase in ASA for interface atoms on going from the U form to the B form for at least one of the two partners, which results from the optimization of contacts through the interface. This change in ASA is independent of whether or not a molecule behaves as a quasi-rigid body or undergoes conformational changes during complex formation. These and other changes that take place in the interface, including optimization of hydrogen bonding to the partner protein, the formation and the extension of regular secondary structures at the cost of turns and coils, reduction of *B* factors (flexibility) and disorder-to-order transitions in the interface residues, presumably contribute to the specificity, the stability and the function of the complex.

## 6. Related literature   

The following references are cited in the Supporting Information for this article: Ratnaparkhi *et al.* (1998 ▸) and Ševčík *et al.* (1998 ▸).

## Supplementary Material

Supporting Information.. DOI: 10.1107/S2052252515015250/lz5009sup1.pdf

## Figures and Tables

**Figure 7:**
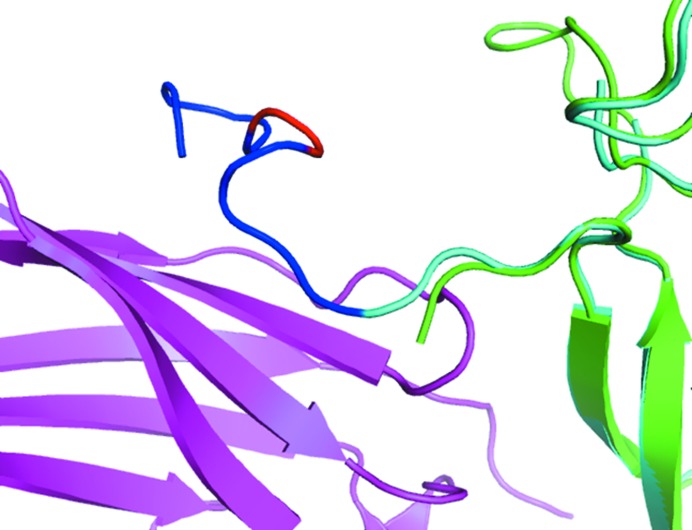
The loop (1–12) missing in the U form of neurotrophin-4 (PDB entry 1b98; Robinson *et al.*, 1999 ▸; shown as green cartoon) which is present in the B form (PDB entry 1hcf; Banfield *et al.*, 2001 ▸; cyan) on forming a complex with the BDNF/NT-3 growth factor receptor TrkB-d5 (magenta cartoon). The interface residues (in blue) are interspersed with non-interface residues (in red) in the missing loop. The contribution of the missing residues is 383 Å^2^ to the BSA of 765 Å^2^.

**Table 1:** Statistics for interface residues missing in the U form and their secondary structure in the B form

		% relative to total No. of		Secondary structure in the B form of residues missing in U† (%)			
Residue	No. missing‡	Interface residues of the same type	Missing residues‡	H	S	T	C
Ala	15 (5)	4.7	7.6 (6.1)	27 (35)	0	13 (10)	60 (55)
Arg	14 (2)	3.2	7.1 (4.9)	28.6 (25)	0	21.4 (18.8)	50 (56.2)
Asn	13 (4)	3.0	6.6 (5.2)	7.7 (5.9)	0	46.2 (41.1)	46.2 (52.9)
Asp	10 (10)	2.3	5.5 (6.1)	50 (30)	0	0 (25)	50 (45)
Cys	1 (2)	0.6	0.5 (0.9)	0	0	0 (33.3)	100 (66.7)
Gln	9 (5)	2.5	4.5 (4.3)	11.1 (7.1)	0	33.3 (50)	55.6 (42.9)
Glu	18 (10)	3.8	9.1 (8.6)	11.1 (14.3)	5.6 (3.6)	27.8 (32.1)	55.6 (50)
Gly	13 (12)	2.5	6.6 (7.7)	0	7.7 (4)	23.1 (40)	69.2 (56)
His	7 (3)	3.2	3.5 (3.1)	0 (10)	0	57.1 (50)	42.9 (40)
Ile	10 (11)	3.5	6.1 (6.4)	10 (9.5)	0 (4.8)	10 (19)	80 (66.7)
Leu	15 (4)	3.3	7.6 (5.8)	33.3 (31.6)	0	6.7 (5.3)	60 (63.2)
Lys	11 (7)	2.3	5.6 (5.5)	27.4 (27.8)	0 (5.6)	36.4 (22.2)	36.4 (44.5)
Met	7 (2)	5.0	3.5 (2.8)	57.2 (66.7)	14.3 (11.1)	28.6 (22.2)	0
Phe	5 (2)	1.9	2.5 (2.2)	0	0	0 (14.3)	100 (85.7)
Pro	11 (9)	3.6	5.6 (6.1)	18.2 (10)	0	9.1 (15)	72.7 (75)
Ser	11 (21)	2.1	5.6 (9.8)	27.3 (12.5)	0 (6.25)	36.4 (37.5)	36.4 (43.8)
Thr	7 (13)	1.5	3.5 (6.1)	28.6 (25)	0 (10)	28.6 (10)	42.9 (55)
Trp	4 (2)	2.6	2.0 (1.8)	0 (16.7)	25 (16.7)	0	75 (66.7)
Tyr	7 (3)	1.6	3.0 (2.5)	0 (10)	0	57.1 (40)	42.9 (50)
Val	9 (4)	2.7	4.5 (4.0)	0	11.1 (15.4)	11.1 (7.7)	77.8 (76.9)
Total	197 (131)		100				

† The numbers in parentheses were calculated considering the entire stretch of missing residues (Supplementary Table S2).

‡ The numbers in parentheses correspond to the non-interface residues in the missing stretch (Supplementary Table S2).
