# Supplementary material for: Changes in protein structure at the interface accompanying complex formation
Source: IUCrJ. 2015 Oct 16;2(Pt 6):643–52. doi: 10.1107/S2052252515015250 (PMC4645109; doi:10.1107/S2052252515015250)
Supplement: Supplementary file 1 [file m-02-00643-sup1.pdf]

IUCrJ

Volume 2 (2015)

Supporting information for article:

Changes in protein structure at the interface accompanying complex formation

Devlina Chakravarty, Joel Janin, Charles H. Robert and Pinak Chakrabarti

Table S1 Details of the comparison between the B and U forms

(a) When residues do not match

PDB file		Residue name
B	U	B/U
1a2k	1qg4	PHE72/TYR72
1e6j	1a43	ALA208/GLY208
1efn	1fyn	ILE96/ARG96
1ezu	1ecz	PHE69/TYR69, PRO70/ASP70
1f6m	1cl0	SER135/CYS135
1fqj	1fqi	MET394/LEU395
1fqj	1tnd	ALA231/VAL231, GLU234/ASP234
1jmo	2cn0	ALA195/SER195
1kkl	2hpr	MET51/ VAL51
1klu	1ste	SER43/LYS43, PHE45/LEU45, LYS46/ALA46, TRP47/HIS47
1lfd	5p21	LYS231/GLU31
1m10	1m0z	VAL239/MET239
1nw9	1jxq	ALA316/GLY287, THR317/GLU288, PRO318/GLN289, SER333/VAL296
1oph	1qlp	ARG358/MET358
1oph	2ptn	ALA195/SER195
1ppe	1lu0	MET8/LEU8
1pxv	1x9y	ALA243/CYS243
1qa9	1ccz	SER85/THR85
1r6q	2wq9	TRP7/GLN12, PHE10/ GLU15, ASP11/GLU16, GLN12/LYS17, LEU13/VAL18
1zli	1kwm	ASN14/ LYS14
2btf	1ijj	VAL287/ILE687, SER365/ALA765
2c0l	1c44	ASN140/LYS120
1i2m	1qg4	PHE72/TYR72
1brs	1a19	ALA40/CYS40
2wpt	1fsj	CYS95/ GLY95
2wpt	2no8	ALA23/CYS23
3sgb	2ovo	LEU18 /MET18

(b) Modified residues (in the whole structure)

PDB file		Residue name
B	U	B/U (with modified residue #)
1f34	4pep	SEP(68)/SEP(68)
2mta	2bbk	TRQ(57)/TRQ(57)
1s1q	1ubq	MSE(1)/MET
1s1q	2f0r	MSE(11,53,95,131)/MET
2btf	1iij	HIC(73)/HIS
1ib1	1kuy	TPO(31)/THR
1atn	1ijj	HIC(73)/HIS
1jmo	1jmj	TYS(60,73)/Missing in U
1zm4	1n0v	DDE(669)/HIS
1bvn	1pig	GLN/PCA(1)
2sni	1ubn	CYS/SOC(221)
2hqs	1crz	MET/MSE(182,252,325,327,367,376)
2oob	2ooa	MET/MSE(940)
1kkl	1jb1	MET/MSE(139,214,282,289)
1xqs	1xqr	MET/MSE(83,93,104,134,146,236,239,271,273)
1eaw,1cbw,2ptc,2tgp	9pti	MET/MHO(52)
2pcc	1ycc	LYS/M3L(72)
1fqj	1fqi	MET/MSE(291,370,394,396,413)
1zhi	1z1a	MET/MSE(528,532,589)
1kkl	2hpr	SER/CSO(83)
3bp8	1z6r	MET/MSE(54,167,176,201,286,289,329,384,394)
3cph	1g16	MET/MSE(21,94,137)

MSE: selenomethionine, SEP: phosphoserine, TRQ: propionic acid, HIC: 4-methyl-histidine, TPO: phospothreonine, TYS: o-sulfo-l-tyrosine, DDE: {3-[4(2-amino-2-carboxyl-ethyl)-1h-imidazol-2-yl]-1-carbamoyl-propyl}-trimethyl-ammonium, PCA: pyroglutamic acid, SOC: dioxyselenocysteine, ASK: dehydroxymethylaspartic acid, MHO: s-oxymethionine, M3L: n-trimethyllysine, CSO: s-hydroxycysteine, ALC: 2-amino-3-cyclohexyl-propionic acid. In 1nw9/1jxq, 1ib1/1qjb and 2i9b/1ywh pairs, the U form has modified residues ASK, SEP and ALC respectively, in chains which are not being compared to the B form.

(c) Missing interface residues/atoms in the U form

PDB file		Residues in B missing in U
B	U	Residue name (atom labels)
1efn	1avv	ARG71*,PRO72*,GLN73*
1e6e	1cje	ASP113*,ARG115*,GLU116*,SER117*
1f34	1f32	GLN1*,GLN118*,GLU119*,ASN120*,GLN121*,PRO133*,ALA134*,LEU137*
1eer	1ern	SER135*
1f6m	1cl0	SER135*
1fqj	1tnd	ALA231*,GLU234*
1gcq	1gcp	SER592*,HIS593*
1grn	1rgp	ALA426*,LYS427*,ALA429*,ALA430*,THR432*,LEU433*,ASN437(OD1),GLU333(OE2),ILE436(CG1,CD1,CG2)
1gxd	1br9	LYS185*,GLN186*,GLU187*,PHE188*,LEU189*,ASP190*,GLU192*
1h1v	1ijj	PHE375*,GLU167(OE2,OE1,CD,CG),LYS315(CG,CE,CD)
1hcf	1b98	GLY1*,VAL2*,SER3*,GLU4*,THR5*,ALA6*,SER9*,ARG10*,ARG11*,GLY12*,ARG53(NE,NH1,NH2,CG,CZ,CD)
1he8	1e8z	LYS255*,SER257*,LEU258*,VAL223(NZ,CE)
1ib1	1kuy	GLY19*,PRO21*,GLY22*,GLN27*,ARG28*,ARG29*,THR31(P,O2,O3,O1)
1j2j	1oxz	ILE168*,PHE169*
1nvu	2ii0	HIS750*,ASN751*,ILE752*,THR753*
1pvh	1emr	ALA13*,ILE14*,ARG15*,HIS16*,PRO17*,CYS18*,HIS19*,ASN21*
1t6b	1acc	GLU343*,ARG344*,GLU348*,THR349*

1xd3	1uch	ALA152*,GLU154*,GLN156*,THR157*,GLU158*,ALA159*,PRO160*,VAL166*
1xqs	1xqr	MET134*
1z0k	1yzm	GLU441*,GLY442*,TRP443*,LEU444*,PRO445*,LEU446*,SER447*,GLU454*,ARG478(NH1),ASP480(OD2,CG),GLN499(NE2)
1yvb	1cew	ARG6*,LEU7*,LEU8*
1zhi	1z1a	MET528*
2a9k	2c8b	ALA247*,ILE248*,ASN249*,PRO250*
2btf	1ijj	PHE375*,GLU167(OE2,OE1,CD,CG),GLU364(OE2,OE1,CD,CG),LYS373(CD,CE,NZ,CG)
2c0l	1c44	LEU143(OXT)
2hqs	1oap	LEU174*
2hqs	1crz	MET204*,VAL350*,MET398*
2hrk	2hqt	HIS123*
2oob	2ooa	ASN931*,MET940*
2oza	3hec	GLY33*,ALA34*,TYR35*,LEU171*,GLY181*,TYR182*,VAL183*
2oza	3fyk	TYR228*,TYR229*,VAL230*,ALA231*,PRO232*,GLU233*,VAL234*,LEU235*,GLY236*,PRO237*,ASN266*,HIS267*,GLY268*,LEU269*,ARG280*,MET281*,VAL365*,ASP366*,TYR367*,GLN369*,ILE370*,LYS371*,ILE372*,LYS373*,LYS374*,ILE375*,GLU376*,ASP377*,ALA378*,SER379*,ASN380*,PRO381*,LEU382*,LEU383*,LYS385*,ARG386*,ARG387*,LYS389*,ALA390*
2pcc	1ycc	LYS73*
2sni	1ubn	SER221*
3cph	3cpi	THR5*,ILE6*,ARG445*
3cph	1g16	ILE53*,GLY54*

1avz	1avv	THR71*,PRO72*,GLN73*
2gox	2gom	ALA103*
3bzd	3bvz	ASN100*,VAL101*,TRP102*,HIS104*
1jmo	1jmj	GLU56*,ASP57*,ASP58*,ASP59*,TYR60*,ASP72*
1jmo	2cn0	ASN147D*,VAL147E*,TRP147A*,ALA147C*,GLY148*,LYS149*,ARG75(NH2,CG,CD)
1a2k	1oun	ASN125*,PHE126*,GLY127*
1jps	1tfh	TRP158*,SER163*
1k5d	1yrg	GLU345*
1klu	1ste	SER96(OG)
1kkl	1jb1	ASN308*,GLU309*, A chain, [GLU204(OE2,CD,OE1,CG),HIS140(CD2,NE2,ND1,CG,CE1)], C chain [ASP240(OD2),GLU298(OE2,CD,CG)]
1ijk	1fvu	Chain C [ASP288*,ASP289*,TYR291*], Chain B [TYR45(CZ,CG,CD2,CD1,CE2,OH,CE1)]
1rlb	2pab	ASN124*,GLU127*
1ezu	1ecz	A chain [PHE69*,PRO70*]
1fqj	1fqi	LEU395*,MET396*
1atn	1ijj	ARG62(NH2,NH1),GLN41(CG),GLU57(OE2),VAL45(CG2,CG1)
1avx	1ba7	ARG563(CD,CZ,NH2,NE,NH1),ARG565(NE,CZ,NH1,NH2,CD)
1e96	1mh1	ILE33(CD1),VAL.36(CG2)
1efn	1fyn	GLU94(OE2,OE1,CD)
1bvk	3lzt	LEU129(OXT)

1de4	1a6z	LEU63(CD2,CD1)
1avz	1fyn	ARG96(CG,NH2,NE,CD,CZ,NH1),GLU94(OE2)
1gla	1f3z	GLU72(CG,OE1,CD),LYS99(CG,CD)
1gpw	1k9v	ARG22(CD,NE,NH2,NH1,CZ),LYS184(CE,CD,CG,NZ),SER183(OG)
1hia	1bx8	LYS34(CE)
1i4d	1mh1	ILE33(CG2),VAL36(CG2,CG1)
1ibr	1qg4	LYS134(NZ)
1ijk	1auq	LYS660(CD,CG,NZ)
1kxp	1ijj	GLU167(OE2,CD,CG,OE1),LYS291(CE,NZ,CG,CD)
1rv6	1fzv	ASN73(OD1,),GLU72(OE1)
1us7	2fxs	LYS102(NZ,CG)
1vfb	8lyz	LEU129(OXT)
1kkl	2hpr	LYS40(CE,CD,NZ)
1xqs	1s3x	LYS250(CD,CG,CE,NZ)
1xu1	1u5y	VAL165(CG2)
2fju	1mh1	VAL36(CG2,CG1)
2hle	2bba	GLN52(OE1,CD,NE2)
2i9b	1ywh	GLN131(CG)
2o3b	1zm8	ARG156(NH2,NH1,CG,NE,CD,CZ),GLU92(OE2)
2pcc	1ccp	GLU35(OE2)
2vdb	3cx9	LYS317(NZ,CD,CE)
3bp8	1z6r	ARG38(NE,NH2,CZ,NH1,CD)

3bp8	3bp3	GLY406(OXT)
4cpa	1h20	VAL38(OXT)
1h1v	1p8x	ASN647*,LYS648*,ILE649*,GLY650*,ARG651*,GLU655*

*The whole residue is missing. Only these residues are used in Table 1.

Residues mentioned in (a) and (c) were not considered during calculation of ASA.

Table S2 Peptide segments (with both interface and non-interface residues) as seen in the B form, but missing in U

PDB_chain (U form)	Residue range and sequence (corresponding to the B form)
1tfh_B	158 -163, **W***KSSS***S**
2pab_A	124 -127, **N***PK***E**
2hqt_A	122 -123, *N***H**
2ooa_A	929 - 931,[a]*LE***N**
2gom_A	101-104,[a]*TD***A***T*
1oun_B	125 - 127,[a]**NFG**
1cje_D	113 - 117,[a]**D***A***RES**
1avv_A	71 - 73,[a]**RPQ**
1f32_A	118 - 121, **QENQ ;** 133 - 137, **PA***GG***L**
1gcp_C	591 - 593,[a]*G***SH**
1rgp_A	426 - 433, *A***KD***A***A***I***TL**
1br9_A	183 - 192,[a] *PP***KQEFLD***I***E**
1p8x_A	646 - 655, *S***NKIGR***FVI***E**
1b98_A	1 - 12, **GVSETA***PA***SRRG**
1e8z_A	255 - 268, **K***K***SL***MDIPESQSEQ*
1kuy_A	18 - 30,[a]*S***G***I***PG***SPGR***QRR**
1fvu_B	288 - 291, **DDY***Y*

1oxz_A	168 - 170, [a]**IF***E*
1jmj_A *	54 - 60 , [a]*GE***EDDDY**; 72 - 94, **D***YIDIVDSLSVSPTDSDVSAGNI*
2cn0_H	147 (**A***B***CDE**) - 149, **W***T***ANVGK**
1jb1_E	308 - 310, [a]**NE***E*
1emr_A	12 - 21,[a] *C***AIRHPCH***N***N**
1acc_A	343 - 350, **ER***TWA***ET***M*
1uch_A	147 - 166, *THETS***A***H***E***G***QTEAP***SIDEK***V**
1cew_I	6 - 8,[a]**RLL**
1yzm_A	441 - 455,[a]**EGWLPLS***GGQGQS***E***D*
2c8b_X	246 - 251,[a]*T***AINP***K*
3hec_A	33 - 37, **GAY***GS;* 170 - 172, *G***L***A;* 181 - 183, **GYV**
3fyk_X	217 – 238, *HNSLTTPCYTP***YYVAPEVLGP***E*; 266 – 269, **NHGL;** 278-281,*RI***RM;** 365 – 390,[a]**VDYEQIKIKKIEDASNPLLLKRRKKA**
3cpi_G	5 – 6,[a]**TI;** 444 – 445,[a]*Q***R**
1g16_A	48 – 54, *SFITT***IG**
2ii0_A	750 – 753, **HNIT**
1avv_A	71 – 73,[a]**TPQ**
3bvz_A	96 – 104, *SSKD***NVW***W***H**

The sequence is given in one-letter code; the interface and non-interface residues are marked in bold and italics, respectively. Only the segments with at least two residues are considered (34 cases).

[a] The segment is at the termini of the protein chain.

***** The structure of residues in the range 83-93 may not be very reliable, as mentioned in the PDB file.

Table S3 Average B-factors for interface and surface residues in B and U states

Residue	Bound		Unbound	
	Interface	Surface	Interface	Surface
Ala	-0.33	0.13	0.04	0.04
Arg	-0.29	0.13	0.01	0.03
Asn	-0.21	0.24	0.08	0.2
Asp	-0.09	0.40	0.31	0.31
Cys	-0.13	0.15	-0.03	0.03
Gln	-0.2	0.25	0.08	0.2
Glu	-0.13	0.38	0.17	0.31
Gly	-0.24	0.25	0.23	0.27
His	-0.25	0.12	0.01	0.04
Ile	-0.28	0.01	0.04	-0.04
Leu	-0.31	0.11	0.04	-0.01
Lys	-0.16	0.23	0.14	0.17
Met	-0.11	0.15	0.11	0.08
Phe	-0.31	-0.09	-0.04	-0.13
Pro	-0.23	0.24	0.17	0.1
Ser	-0.27	0.32	0.12	0.23
Thr	-0.31	0.19	0.03	0.06
Trp	-0.25	-0.13	-0.11	-0.21
Tyr	-0.27	-0.1	-0.04	-0.23
Val	-0.33	0.1	-0.06	-0.04

Table S4 Average B-factors for interface rim and core in B and U states

Residue	Bound		Unbound	
	Core	Rim	Core	Rim
Ala	-0.26	-0.16	0.019	0.034
Arg	-0.24	-0.12	0.07	-0.03
Asn	-0.21	-0.05	0.09	0.05
Asp	-0.13	0.01	0.19	0.24
Cys	-0.1	-0.07	-0.02	-0.01
Gln	-0.19	-0.05	0.1	0.008
Glu	-0.21	0.04	0.07	0.16
Gly	-0.25	-0.06	0.15	0.18
His	-0.23	-0.06	0.008	-0.02
Ile	-0.24	-0.11	0.012	0.02
Leu	-0.24	-0.15	0.04	0.04
Lys	-0.17	-0.09	0.14	0.06
Met	-0.13	-0.002	0.06	0.06
Phe	-0.29	-0.05	-0.04	-0.006
Pro	-0.19	-0.08	0.11	0.11
Ser	-0.26	-0.05	0.06	0.11
Thr	-0.29	-0.12	0.004	0.01
Trp	-0.19	-0.09	-0.07	-0.07
Tyr	-0.30	-0.06	-0.08	0.03
Val	0.22	-0.20	-0.01	-0.04

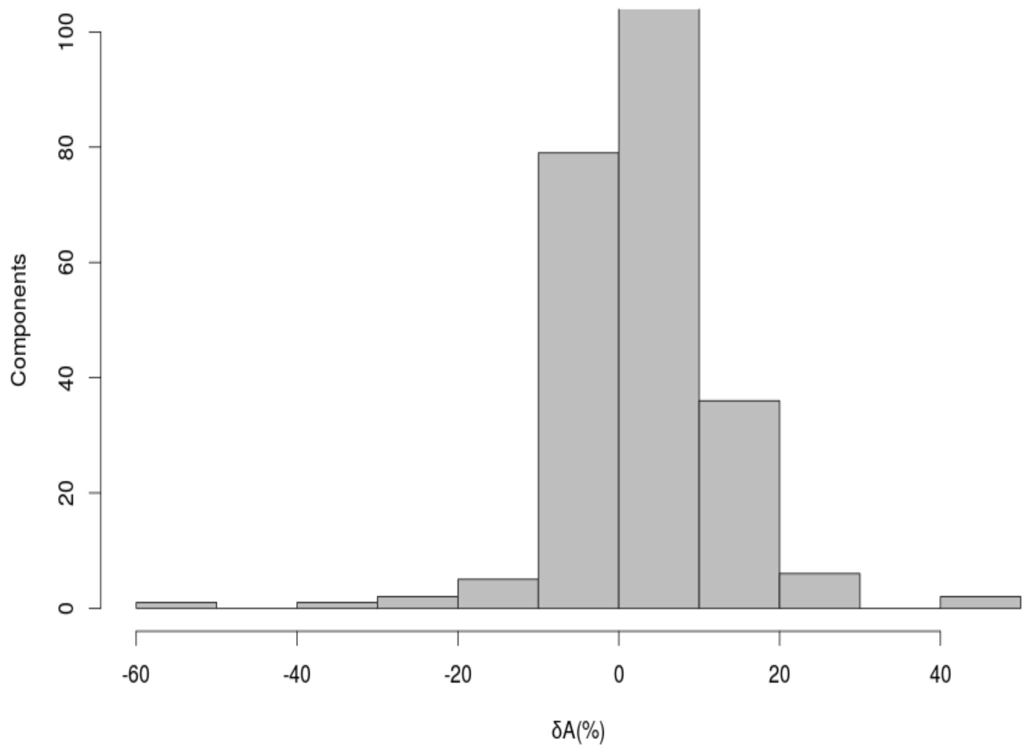

Figure S1 Distribution of δA (%) for 281 components. This differs somewhat from Figure 1 of Chakravarty *et al*. (2013), in which five structures were excluded for reasons specified in that work. Retaining these cases here gave a mean of 3.3 ± 9.2% (as opposed to 3.3 ± 7.2% in the earlier work).

(a)

(b)

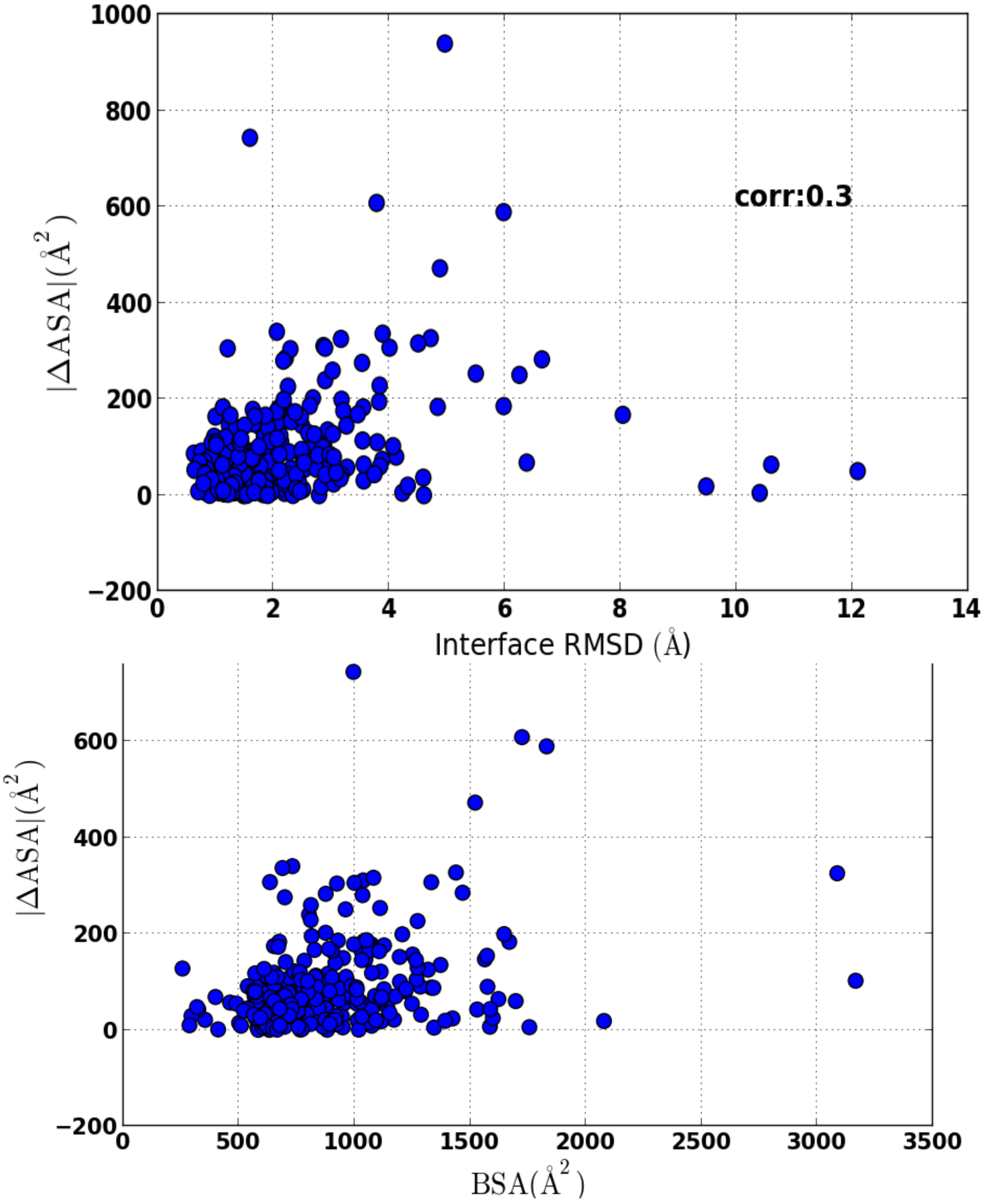

Figure S2 Plot of absolute value of ΔASA (Å^2) vs. (a) interface RMSD (Å) and (b) BSA (Å^2). RMSD is based on all the interface atoms.

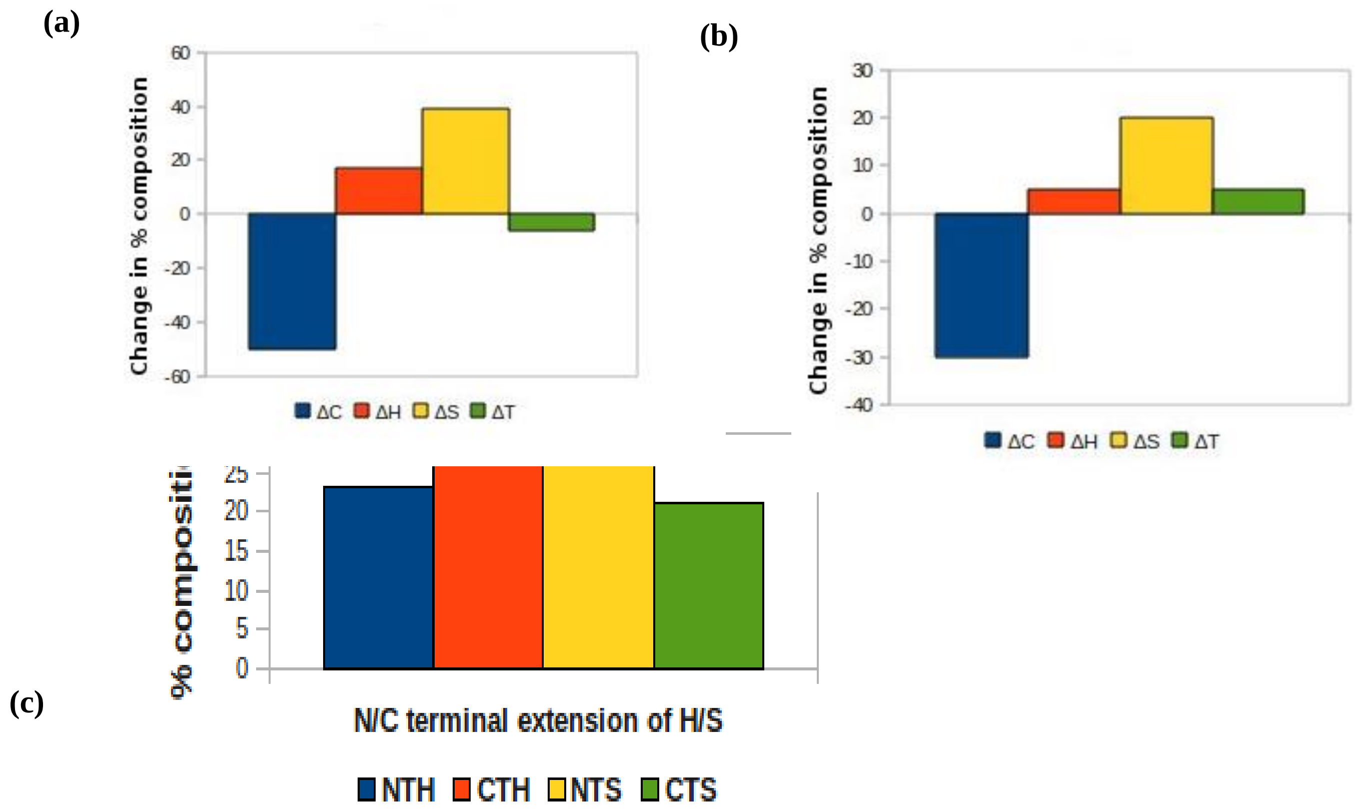

Figure S3 The change in percentage composition between the two states (B - U) for the secondary structural elements for the cases with Euclidean distance between the two sets of compositions being greater than (a) 10 and (b) 15. (c) Percentage of residues involved in the extension of helix or strand are being separated based on their location at the N- or C-terminal end of the respective secondary structural element.

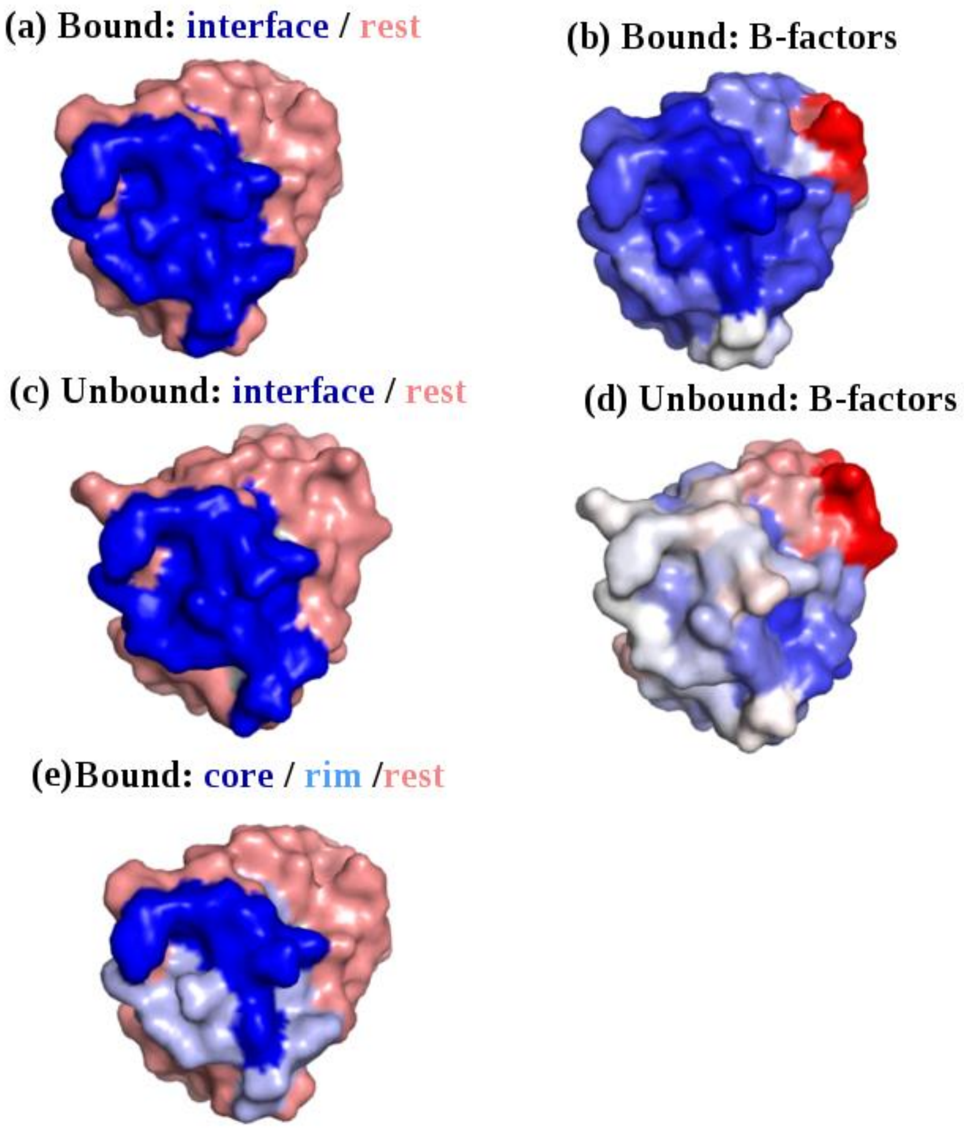

Figure S4 The distribution of B-factors across the interface and the rest of the surface. Shown is barstar in the (a, b, e) bound (1ay7) (Sevcik *et al.*, 1998) and (c, d) unbound (1a19) (Ratnaparkhi *et al.*, 1998) forms, the former being a complex with guanyl-specific ribonuclease (RNaseSa). (a) and (c) dissect the whole protein surface into interface (blue) and the rest (pink) in surface representations; the interface is further divided into core (dark blue) and rim (light blue) in (e). (b) and (d) show the scaled B-factors (the color changes from a value of -1 (blue) to +1 (red)) for the bound and the unbound forms, respectively.
